# Acid Sulfate Soils and Their Pathways of Impact: A Swedish Case Study

**DOI:** 10.1002/ece3.71732

**Published:** 2025-07-10

**Authors:** Lina M. Rasmusson, Nathalie Strokirk, Michael Tedengren, Markus Giese

**Affiliations:** ^1^ Department of Biological and Environmental Sciences University of Gothenburg Gothenburg Sweden; ^2^ Department of Earth Sciences University of Gothenburg Gothenburg Sweden; ^3^ Department of Ecology, Environment and Plant Sciences Stockholm University Stockholm UK

**Keywords:** coastal acidification, estuary, *Hyalella azteca*, Kattegat, metal contamination

## Abstract

Acid sulfate soils (ASS) are naturally occurring sediments rich in iron sulfides that, when exposed to oxygen, produce sulfuric acid, and mobilize toxic metals. Recent findings have identified acidic, metal‐enriched layers in postglacial, organic‐rich sediments in agricultural areas along the Swedish west coast. Although leaching from these soils into a nearby river system has been observed, the extent and downstream consequences remain poorly understood. This study investigates the environmental impact of ASS on a river–estuary system along the Swedish west coast, with a focus on metal mobility and ecological effects. To assess the environmental impact of ASS leachate, water quality parameters (pH and electrical conductivity) and metal concentrations were measured across a river–estuary gradient. Metal concentrations were also analyzed in sediment and biological tissues, and an ecological survey of aquatic flora, fauna, and microbial communities in the sediment was conducted. Additionally, a laboratory toxicity test was conducted on the freshwater amphipod *Hyalella azteca* using riverine water sampled near the ASS site. Results showed that although metal concentrations in water, sediment, and plant tissues remained elevated in the estuary, they declined with distance from the river mouth. Similarly, pH levels were notably lower at the river outflow compared to nearby reference rivers during both spring and autumn. In general, the estuary lacked macrobiodiversity, as low organism abundance of both plants and animals was encountered in the ecological survey. However, a rich microbial community dominated by different orders of cyanobacteria was identified through metabarcoding. Amphipods exposed to river water exhibited signs of developmental stress, including reduced head length, lower instar stages, and decreased dry weight compared to controls. The findings indicate that leaching from ASS leads to acidification and widespread metal contamination, with demonstrable ecological effects that may hinder the achievement of good water status under the European Union Water Framework Directive.

## Graphical abstract

This study is an initial report on the situation in an estuary affected by acidic sulfate soils in an area of Sweden where this has not been investigated before. The study clearly states that metals and acidic effluents from the agricultural acidic sulfate soil are transported via riverine water to the adjacent estuary where metals accumulate in the sediment and biota. This has strong adverse effects on the aquatic flora and fauna, and may impose a risk on several trophic levels.

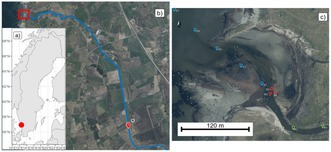

## 1 Introduction

Acid sulfate soils (ASS) are naturally occurring soil types found worldwide (e.g., Dent and Pons 1995). They are often considered among the nastiest soil types due to their potential to generate sulfuric acid, promoting extremely low pH conditions and mobilizing metals (Fanning et al. 2017; Nyman et al. 2024). These sulfur‐rich soils form in waterlogged environments with high organic matter content, where bacterial degradation of organic material creates anoxic conditions (e.g., Asif et al. 2025).

In a reducing environment, these soils remain dormant and are referred to as hypersulfidic soil (Boman et al. 2023). However, exposure to oxygen, induced by e.g., drought (Mosley et al. 2017), excavation work (Kawahigashi et al. 2008), dredging (Johnson et al. 2022), or land uplift (Dalhem et al. 2025), activates these soils, leading to acidification of the soil matrix caused by the oxidation of pyrite and metastable iron sulfides, for example, mackinawite and greigite, and the precipitation of sulfuric acid (Fanning et al. 2017). The soils are then termed sulfuric soils (Boman et al. 2023; Rabenhorst et al. 2025). Certain metals, such as aluminum (Al) and iron (Fe), exacerbate acidity by releasing acidic cations. Soil pH can drop to lower than 2, intensifying weathering and mobilizing metals such as Al, cadmium (Cd), cobalt (Co), copper (Cu), Fe, lithium (Li), manganese (Mn), nickel (Ni), and zinc (Zn) (Ha et al. 2019; Hulisz et al. 2022; Nyman et al. 2024). These metals, many of which are harmful to biota, can leach into nearby streams and rivers (Nystrand et al. 2016; Virtasalo et al. 2020; Lindgren et al. 2022; Shahabi‐Ghahfarokhi et al. 2022; Toivonen and Boman 2024; Firmino et al. 2025). Consequently, rivers draining sulfuric soil areas are characterized by low pH and elevated metal concentrations, with temporary spikes during spring snowmelt and autumn precipitation (Åström and Björklund 1995; Åström 2001; Nystrand et al. 2016). Annual metal releases from ASS areas can exceed industrial discharges by several orders of magnitude (Sundström et al. 2002), and in many cases, they exceed the EU Environmental Quality Standard (EQS) (Toivonen and Boman 2024). Thus, ASS poses a dual environmental threat, combining extreme acidity and high metal concentrations that often act synergistically to cause acute and long‐lasting ecological effects.

Estuarine environments are typically highly productive and biologically active. However, these habitats have a naturally high complexity due to diel or seasonal variations in salinity, tidal range, temperature, and pH (e.g., Day et al. 2012). Organisms adapted to such shifting conditions may already live at their tolerance limits, and the influx of ASS‐derived water can act as a tipping point, reducing species diversity and abundance. Acidic surface runoff can contribute to coastal or estuarine acidification, which can adversely affect calcifying organisms. These effects are comparable to those of ocean acidification, including reduced calcification, dissolution of calcareous structures, and impaired growth (Barton et al. 2015; Fitzer et al. 2018). Additionally, the combination of low pH and high metal concentrations disrupts yolk sac and fish‐larval development, and has geno‐ and cytotoxic properties, leading to higher mortality rates in fish populations (Authman et al. 2015; Pinheiro et al. 2019; Toivonen et al. 2020). Moreover, aquatic invertebrates, important intermediates between producers and higher consumers in the aquatic food web, are sensitive to this combination (Fornaroli et al. 2018; Jeong et al. 2023). Bioaccumulation and biomagnification of metals in ASS‐affected areas occur across trophic levels, from aquatic plants to apex predators (Öhlander et al. 2014; Toivonen et al. 2020; Vainio et al. 2020), posing potential risks to human health (Åström and Roos 2022).

In Sweden and other parts of Fennoscandia, ASS are prevalent in regions below the postglacial highest coastline. The postglacial rebound in this area, one of the highest land uplifts globally (Uścinowicz 2003), has facilitated the ongoing oxygenation and formation of sulfuric soils (Dalhem et al. 2025). Recent discoveries of sulfuric soils in southwestern Sweden have expanded their known distribution to densely populated regions with significant anthropogenic activity (Lindgren et al. 2022; Nyman et al. 2023). In these areas, surface water exhibited low pH and significantly elevated metal concentrations, particularly in agricultural ditches. Gradually increasing pH and decreasing metal concentrations downstream indicated mixing and metal precipitation processes along the way from the source to the ocean (Lindgren et al. 2022). While research has primarily focused on terrestrial impacts, there is a growing need for comprehensive studies on the distribution and effects of ASS in aquatic environments.

The discovery of ASS along Sweden's west coast underscores potential risks to compliance with the EU Water Framework Directive (European Commission 2003), which emphasizes protecting and restoring aquatic ecosystems, addressing both surface and groundwater quality. Acidic drainage containing heavy metals and other contaminants necessitates urgent assessment of ASS impacts on connected aquatic systems.

Two hypotheses were tested to assess the impact of sulfuric soils:

1. Runoff from agricultural ASS contributes metals to water, biota, and sediments in adjacent estuarine and marine environments.
2. ASS‐related runoff adversely affects aquatic biota in the estuary, challenging efforts to maintain biodiversity and ecosystem health.

This study aimed to monitor metal concentrations in water and sediments along a gradient from agricultural sources to estuarine and ocean environments. Key parameters, including pH, electrical conductivity, metal accumulation in biota, sediments, and water, as well as bacterial community dynamics, were measured. Laboratory‐based toxicity tests were also conducted using riverine water near the source to understand ecological impacts. By aligning the study with the EU Water Framework Directive, the findings aim to provide actionable insights to mitigate ASS‐related risks, support sustainable agricultural practices, and preserve aquatic ecosystems.

## 2 Material and Methods

### 2.1 Site Description

**FIGURE 1:**
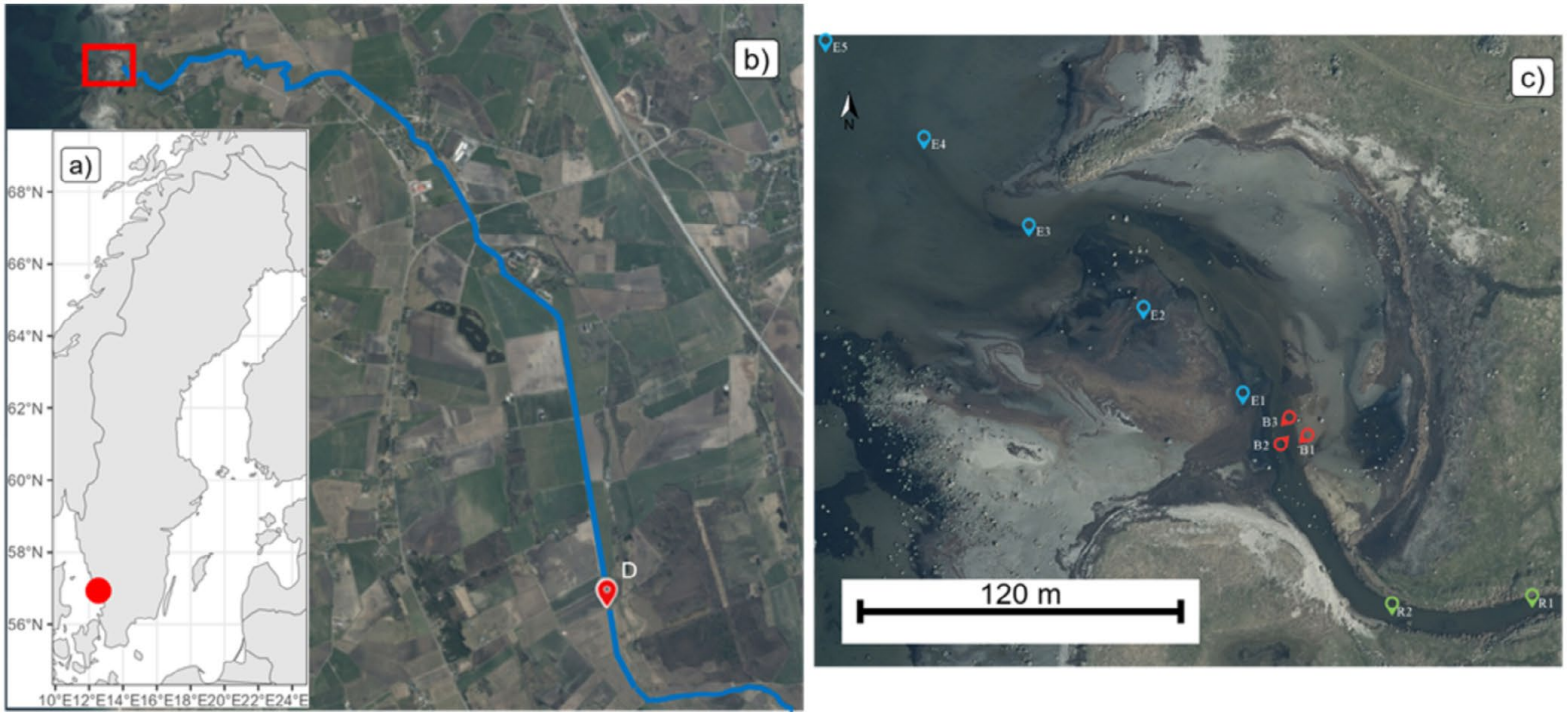
Map of the study area of Ramsjö Canal (b) and estuary (c), Halland county, Sweden (a). Sampling points in agricultural ditch (D), the river (R1–R2), estuary/ocean (E1–E5), and for bacteria/community metabolism (B1–B3). The blue line represents part of Ramsjö Canal.

The study focuses on Ramsjö Canal and its estuarine and oceanic catchment area, located in Falkenberg Municipality, Halland County, Sweden (Figure 1). Ramsjö Canal, a man‐made feature, was created in the mid‐1800s to drain Lake Ramsjö and adjacent boglands for agricultural expansion (Lindgren et al. 2022). The 12‐km‐long canal, with several connecting ditches, drains an area of approximately 60 km^2^, most of which consists of agricultural land. The canal flows into the Kattegat Sea, forming a well‐mixed estuary (56°59′05″N, 12°21′24″E) with an approximate area of 0.016 km^2^ and a mean depth of 0.5 m at low tide. The current moves in a northerly direction before mixing with the ocean.

At this site, acid sulfate soils contain layers that comprise organic‐rich postglacial sediments, including gyttja clay and loamy sand. These soils were likely formed in shallow, postglacial coastal lagoons, resulting in a patchy and complex spatial distribution that complicates mapping and analysis. For a more specific description of the soil properties, including pH profiles and metal concentrations, see Lindgren et al. (2022).

Lindgren et al. (2022) and later Nyman et al. (2023) concluded that the distribution of ASS in this geographical area is likely significantly underestimated. This complicates the identification of adjacent reference sites unaffected by ASS. To address this, pH data from the regional environmental monitoring (RMÖ) program by the County Administrative Board in Halland were assessed to compare Ramsjö Canal with other river outlets in Halland County for the same months in 2021.

### 2.2 Sample Collection and Analyses

Field campaigns were conducted in March (winter), May (early summer), July (summer), and October (fall) 2021. During these dates, point measurements of pH, temperature, and electrical conductivity (EC) were taken, and water and sediment samples were collected for metal content analyses. Additionally, at different time points, samples of aquatic plant tissue (for metal content), infauna (to assess biodiversity), and sediment (for bacterial metabarcoding) were collected. In July, community metabolism was measured, and water samples were collected for laboratory experiments.

An overview of spatial and temporal information, along with the actions conducted, is provided in Table S1. Sample points are highlighted in Figure 1.

#### 2.2.1 Surface Water Samples

Water samples were collected using a 500 mL beaker connected to a telescopic rod. Simultaneously, the temperature (°C), pH, and EC were measured in situ at each site with a handheld multimeter (Hanna HI98194, Bedfordshire, UK). At each site, two 250 mL plastic bottles were filled to the brim with water and stored at a controlled temperature (6°C–8°C) to preserve sample integrity prior to analysis.

Upon returning to the laboratory at the Department of Earth Sciences, University of Gothenburg (GU), the water samples were filtered through disposable 0.45 μm filters (VWR, Radnor, USA) into 25 mL test tubes. A volume of 0.1 mL of 65% HNO_3_ was added to each tube, resulting in an HNO_3_ content of 1.0%. A subsample of 8 mL from each water sample was analyzed using Inductively Coupled Plasma Mass Spectrometry (ICP‐MS) (Thermo Scientific iCAP TQ ICP‐MS, Thermo Fisher, Waltham, USA) to obtain metal content. The concentrations of the following elements were analyzed: aluminum (Al), arsenic (As), cadmium (Cd), cobalt (Co), chromium (Cr), copper (Cu), iron (Fe), manganese (Mn), nickel (Ni), lead (Pb), vanadium (V), and zinc (Zn).

#### 2.2.2 Sediment Samples

Sediment samples were collected at the indicated sampling points, where three subsamples from the top 5 cm layer of the sediment were taken using a plastic tube (ø 64 mm) and pooled together in a plastic bag. From the pooled sample, a subsample of 50 mL was extracted for metal content analysis. The samples were sent to an accredited laboratory (ALS, Luleå, Sweden) for analysis.

The samples were dried at 50°C and then ground to ensure uniformity. They were digested with 7 M nitric acid in a hot block and analyzed in accordance with SS‐EN ISO 17294‐2:2016 and US EPA Method 200.8:1994 after digestion according to protocol S‐PM59‐HB. The concentrations of the following elements were analyzed: Al, As, barium (Ba), Cd, Co, Cr, Cu, Hg, Ni, Pb, V, and Zn using inductively coupled plasma spectroscopy (ICP‐SFMS).

#### 2.2.3 Biological Samples

To obtain plant tissues, submerged rocks were scraped to collect overgrowths of filamentous green algae. Plant material was only found at sites E1–E3. The collected tissue was placed in plastic bags, transferred to Falcon centrifuge tubes, and then stored in a refrigerator. Due to the poor condition of the tissue, it was impossible to separate individual strands for visual species identification (most likely *Cladophora* spp.). The tissue is referred to as “filamentous green algae” throughout the manuscript.

Plant tissue samples were sent to the accredited laboratory ALS, Luleå. Determination of metals was conducted using ICP‐SFMS according to the standard SS‐EN ISO 17294‐2:2016 and the US EPA Method 200.8:1994 after digestion according to B‐PB29‐MW. Concentrations of Al, As, Cd, Co, Cr, Cu, Hg, Mn, Ni, Pb, and Zn were measured.

For infauna sampling, three sediment samples were randomly collected within a 5 m radius from the midpoint of each site (E1–E5; Figure 1). The top 5 cm sediment layer was collected using a plastic sample tube (ø 64 mm). The sediment was sieved through a 2 mm mesh, and the retained fauna was transferred to 50 mL Falcon centrifuge tubes containing 40 mL of 95% ethyl alcohol. The samples were refrigerated (6°C–8°C) until identification. Species determination was undertaken at Stockholm University (SU), where specimens were classified to the family or order level. However, due to insufficient biomass, no metal content in infauna was analyzed.

For microbial community assessment, sediment samples were collected from three sites (B1–B3) near the river outlet (Figure 1). The top 2 cm sediment layer was collected using a plastic tube until a volume of 15 mL was obtained. Samples were placed on ice in a cooler box, transported to SU, and stored at −20°C until analysis.

In addition, transects using an aquascope were carried out throughout the estuary to visually determine flora and fauna.

### 2.3 Benthic Community Metabolism

The benthic community metabolism was measured in situ using benthic chambers at sites B2 and B3 (Figure 1), with two chambers deployed per site, for a total of four chambers. Clear acrylic chambers (Ø 30 cm, h 30 cm, and volume 21.2 L) were inserted 3 cm into the sediment, thus giving a water volume of 19.1 L. An oxygen sensor connected to a portable multimeter (Hach HQ40D, Ames, USA) was used to measure the initial dissolved oxygen (DO, mg/L) and water temperature (°C) in each chamber. The probe was inserted through a hole in the chamber lid, which was then sealed with a rubber stopper after the measurement.

Two enclosures (one per site) were darkened using black plastic to measure community respiration (R), while the remaining two enclosures were left uncovered and exposed to light to measure community net photosynthesis (NP). The enclosures were incubated for 30 min, after which the final DO concentration and water temperature were recorded from each enclosure. The difference in O_2_ concentration over time was used to calculate respiration (R) and net primary production (NPP) after including time, water volume, and surface area in the equation. Gross primary production (GPP) was calculated as GPP = NP + *R* during the light period.

Based on the molar weights of oxygen and carbon, a factor of 0.375 was used to convert production and respiration rates to carbon, and all community metabolism values are expressed as g C/m^2^ per day.

### 2.4 Metabarcoding Estuarine Microbial Communities

#### 2.4.1 DNA Extraction and Sequencing

Sediment samples were thawed, centrifuged at 3000 *g* for 5 min to facilitate the removal of RNAlaterTM stabilization solution. DNA was then extracted from 2.9 ± 0.7 g of the sediment using the DNeasy PowerMax Soil DNA Isolation Kit (Cat# 12988–10; QIAGEN, Hilden, Germany). Following this procedure, the DNA in these samples was frozen at −20°C in 5 mL of C6 elution buffer (10 mM Tris). DNA extracts were then shipped to Novogene (Cambridge, UK) for DNA normalization, library preparation, and sequencing. DNA concentration was normalized by Novogene according to their in‐house company protocols. Amplification of the 16S rRNA gene V4 region was conducted using the primers 515 F and 806 R (Apprill et al. 2015; Parada et al. 2016). Library preparation was conducted using the NEBNext Ultra II DNA Library Prep Kit with index adapters synthesized in‐house by Novogene. The pooled library was sequenced on the Illumina NovaSeq 6000 SP platform with a 2 × 250 bp paired‐end setup. The raw sequencing data have been uploaded to Figshare (see Data Availability Statement).

#### 2.4.2 Bioinformatics

Raw reads were analyzed according to the DADA2 (version 1.28) pipeline (Callahan et al. 2016) with default settings except for the filter commands: truncLen = c(0,0), maxEE = 2, truncQ = 2, and trimLeft = c(22,21); error model: MAX_CONSIST = 30; merging of pair‐ends: minOverlap = 10; and chimera removal: allowOneOff = TRUE and minFoldParentOverAbundance = 4. After trimming, filtering, merging pair‐ends, and chimera removal, 154,780 sequences were retained, averaging 51,583 reads per sample (minimum 42,415 and maximum 66,048 reads per sample).

In total, 10,569 individual amplicon sequence variants (ASV) were identified. The sequences in the created ASV table were then assigned to taxonomy using the SILVA database version 132 (Quast et al. 2013) and analyzed further as relative abundances (i.e., [counts/∑counts] × 100) in RStudio software (R Core Team 2020).

### 2.5 Laboratory Experiments

Toxicity tests were conducted in a controlled laboratory setting at the Department of Ecology, Environment and Plant Sciences (SU) to assess potential harmful effects on aquatic life, here represented by freshwater amphipods ( *Hyalella azteca* (Saussaure, 1858)), between July and August 2021. The standardized biological test method EPS 1/RM/33 (ECCC 2017) was used to monitor survival, reproduction, and growth. *H. azteca* has a short life cycle, reaching maturity within 23–33 days at 25°C. Their high laboratory tolerance and sensitivity to toxicity make them ideal model organisms for such tests (e.g., Cooper 1965).

Test organisms were collected from a laboratory culture, consisting of two identical (25 L) aquaria filled with dechlorinated tap water (JBL Biotopol, 0.25 mL/L). Each aquarium contained five gauze pieces (approximately 5 × 5 cm each) as a substrate substitute on which the *H. azteca* can attach. Using a stainless tweezer, three to four gauze pieces were picked up and placed in a 500 mL glass beaker filled with chamber water, and the gauze pieces were gently scraped with the tweezer, collecting any attached test individuals into the beaker.

To obtain organisms of preferable age (10 + −1 days, second—to third instar) (ECCC 2017; Lotufo and Farrar 2018), the collected *H. azteca* were gently poured over two connected sieves (⌀ 20 cm) with a mesh size of 800 μm (top) and 500 μm (bottom) and placed over a glass Petri dish (⌀ 20 cm). Subsequently, individuals older than 10 ± 1 days were detained in the 800 μm sieve, while the 500 μm sieve collected individuals of preferred age. Younger individuals landed in the Petri dish. Specimens of undesired age were returned to the original culture aquaria. *H. azteca* from the 500 μm sieve were counted and transferred into an aerated 1000 mL glass beaker, containing chamber water and 5 × 5 cm^2^ gauze pieces, later placed in a thermostat room, set at 24°C overnight. The number of collected individuals was > 350.

Eight 400 mL glass beakers were washed thoroughly with deionized water before being filled with water, three beakers with water from site D (Figure 1) and five with water from a small freshwater pond in Norra Djurgården, Stockholm, a site with no known ASS connection. In each beaker, a 5 × 5 piece of gauze was placed as a settling medium for the animals. The beakers were placed in a thermostat room set at 24°C, and each beaker was aerated with an airflow of approximately one air bubble per second, as described by the ECCC‐Protocol (2017). Light was provided from luminescent tubes (L 36 W/530 Warm White, Osram, Sweden) with a 16/8 cycle, light and dark, respectively. A layer of non‐respiring quartz sand (Rådasand, Lidköping, Sweden) was placed in each beaker as the bottom substrate. After approximately 12 h, the desired temperature of 24°C and air saturation of at least 80% (sensu ECCC, 2017) were reached, and the initiation of the experiments started. In each beaker, 20 randomly chosen individuals of *H. azteca* were placed, 160 in total, 60 individuals for the contaminated and 100 individuals for the control. Individual counts were done every 24 h, and any dead *H. azteca* were removed. Following the same interval, temperature and oxygen saturation were measured in each beaker to ensure optimal conditions. The experimental setup ran for 42 days. After this period, the survival rate (%), head length (mm; sensu Kokkotis and McLaughlin 2002), growth rate (mg dry weight/individual; sensu, ECCC 2017), instar (developmental state; sensu Kokkotis and McLaughlin 2002), number of antenna segments (sensu Geisler 1944), and sex ratio was determined (number of males/females). During the experimental period, the amphipods were fed with ground Tetramin fish food flakes mixed with water three times per week according to the test protocol ECC (2017).

The data were processed statistically using Microsoft Excel for Microsoft 365 MSO (Version 2411). Welch's t‐test was utilized to determine statistically significant differences in mean values between the two groups for the following variables: head length, dry weight, instar stage, survival rate, antenna segment count, and sex ratio.

## 3 Results

In all graphs, sampling point D has been included to compare conditions in one of the agricultural ditches with those in the estuary and ocean. This particular ditch collects the majority of runoff from ASS before it enters Ramsjö Canal (sensu Lindgren et al. 2022).

### 3.1 Measurements of Water Quality

**FIGURE 2:**
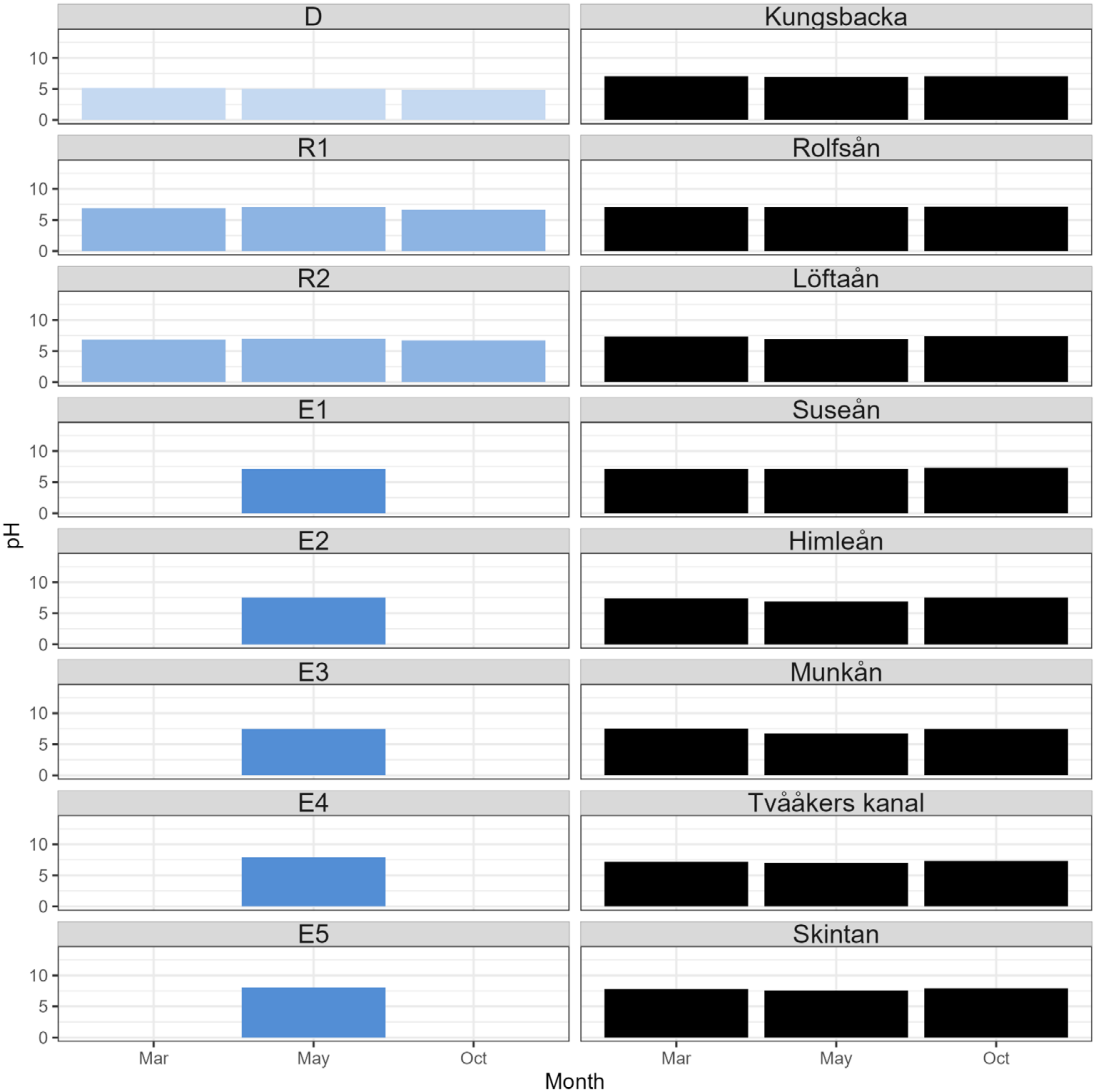
pH values in water for all sample points for Ramsjö Canal, as well as from other riverine outlets in the region, were measured by the county administrative board in Halland. The lowest pH measured is from the agricultural acid sulfate soil ditch (D), followed by a low pH in the connected canal (R1–R2) and estuary system (E1–E5). A lower pH level is recorded in the Ramsjö river outlet in spring and fall compared to other rivers in the region.

Point measurements from three different seasons (winter, early summer, and fall) did not show any obvious seasonal variations in pH or EC, as results from all sampling sites were similar during each round (Figure 2; Table S2). The lowest pH was measured at D (early summer: pH 5.0, fall: pH 4.8), and pH increased with distance from the ASS, with the highest value observed at E4 (pH 7.9) (Figure 2; Table S2). Point measurements of river outlets provided by the County Administrative Board of Halland showed that pH was substantially lower in March and October at the Ramsjö Canal outlet (Figure 2). In May, the pH at Ramsjö Canal was similar to or even higher than that of the other rivers in Halland County. EC was relatively stable in the estuary, except at E4 and E5, which displayed an oceanic influence, with values as high as 15,000 and 22,000 μS/cm (Table S2). The temperature naturally varied with the season, but no specific differences were observed between sampling points (Table S2).

### 3.2 Metal Concentrations in Water, Sediment and Tissue

The concentrations of all metals were highest in the agricultural ditch (D), where Al, Co, Cr, Cu, Mn, Ni, and Zn exceeded regional (Herbert et al. 2009) and national background values (Edén and Björklund 1993; Naturvårdsverket 2008). Even though concentrations were highest in D, metal content in water and sediment remained high, and in many cases exceeded reference values in the river (R1–R2) and estuary (E1–E5) water. During winter (March), concentrations in water were lower than in early summer (May) and fall (October). However, during this time, the sediment content of these metals was much higher than later in the year (Table 1a,b).

**TABLE 1:** Metal content in water (a) and sediment (b) from all sample points, and filamentous algae tissue (c) collected from sites E1–E3.

Site	Month	Al	As	Ba	Cd	Co	Cr	Cu	Fe	Hg	Mn	Ni	Pb	V	Zn
**(a)**															
D	March	**936.6**	0.28	—	b.d.l.	**1.1**	**0.68**	**2.9**	51.1	—	2.4	**11.6**	0.17	0.41	**84.7**
D	May	**1974.9**	0.27	—	0.0021	**15.2**	**0.82**	**12.0**	152.4	—	**1011.3**	**26.0**	0.20	0.21	**76.2**
D	Oct.	**2709.1**	0.31	—	0.0022	**17.4**	**1.12**	**16.1**	607.6	—	**1104.9**	**46.8**	**2.06**	0.17	**469.3**
R1	March	**420.5**	0.34	—	b.d.l.	**1.5**	0.48	**5.1**	463.1	—	**151.5**	**5.2**	0.22	**0.87**	**22.8**
R1	May	21.2	0.24	—	0.00149	**1.5**	0.22	**49.4**	24.9	—	**134.6**	**4.4**	0.04	0.48	3.9
R1	Oct.	37.5	0.29	—	0.00150	**2.4**	0.33	**28.6**	57.0	—	**194.7**	**9.0**	**2.05**	0.59	**66.3**
R2	March	150.2	0.29	—	b.d.l.	**1.3**	0.29	**3.0**	133.2	—	**137.9**	**4.4**	0.17	0.46	**18.5**
R2	May	**355.4**	0.29	—	0.0020	**1.6**	0.40	**90.3**	332.0	—	**137.5**	**5.2**	0.24	**0.80**	**11.8**
R2	Oct.	30.4	0.28	—	0.0010	**2.3**	0.31	**5.3**	57.7	—	**195.0**	**7.1**	0.33	0.52	**26.1**
E1	May	16.5	0.28	—	0.0010	**1.2**	0.19	**20.8**	17.1	—	**112.2**	**4.1**	0.05	0.45	3.9
E2	May	17.3	0.30	—	0.0014	**1.1**	0.20	**4.0**	18.1	—	**82.7**	**3.9**	0.03	0.52	2.3
E3	May	51.5	0.25	—	0.0017	**1.4**	0.29	**35.1**	37.9	—	**122.7**	**4.6**	0.03	0.44	6.6
E4	May	55.5	**0.42**	—	0.0103	**1.1**	0.32	**30.9**	28.7	—	**104.8**	**3.6**	0.09	0.47	**7.9**
E5	May	48.3	0.35	—	0.0065	**1.3**	0.23	**28.8**	29.5	—	**123.5**	**4.0**	0.05	0.50	**7.6**
RV		228	0.39		0.027	0.29	0.6	1.1	771		61	0.76	0.5	0.7	6.8
**(b)**															
D	May	32,700	11	69.5	0.83	11.2	26.9	132	—	< 0.2	—	22.7	20.9	50.2	106
R1	May	13,800	7.98	80.4	3.13	20.3	12.9	27.3	—	< 0.2	—	31.8	14.5	34.2	280
R2	May	1770	0.66	18.6	0.12	3.61	1.73	2.05	—	< 0.2	—	2.41	2.1	8.7	21.4
E1	May	1550	0.61	8.2	0.14	2.13	1.7	1.58	—	< 0.2	—	2.39	2.2	5.1	16.4
E2	May	860	< 0.5	6.4	< 0.1	0.9	1.04	0.82	—	< 0.2	—	0.87	2.3	3.1	10.1
E3	May	932	0.56	6.2	< 0.1	1.46	0.801	1	—	< 0.2	—	1.06	< 1	3.0	10.2
E4	May	973	0.73	8.5	< 0.1	1.18	1.18	0.87	—	< 0.2	—	1.26	1.6	3.2	10.5
E5	May	870	0.88	7.2	< 0.1	1.14	1.44	0.70	—	< 0.2	—	1.08	1.5	3.8	9.3
**(c)**															
E1	May	7690	2.6	—	0.71	18.5	8.02	10.4	—	0.02	1470	15.4	4.6	—	126
E2	May	7140	3.09	—	1.04	15.9	14	15.5	—	0.03	995	19.8	4.43	—	130
E3	May	4680	3.31	—	1.39	12.5	6.72	14.9	—	0.04	821	14.46	3.44	—	113

*Note:* Values indicate μg/L (water samples) or mg/kg dry weight (sediment and tissue) of the metals. RV in water sample table a = regional reference value according to Herbert et al. (2009), concentrations measured above this value are indicated in bold letters. b.d.l., below detection limit. Site descriptions in Table S1.

Concentrations of As, Cd, Pb, and V were not considerably higher in the agricultural ditch than in the estuary, and the concentration spikes observed for some of these elements were not associated with a specific sampling point in the system (Table 1a). However, none of these metals, except Cd, is known to be related to ASS. Plant tissue collected from sites E1–E3 contained high concentrations of most metals examined, particularly Al and Mn, but also Co, Cr, Ni, Pb, and Zn (Table 1c). For most metals in all phases (water, sediment, or tissue), metal content decreased with distance from the river outlet. However, Al, Cu, Mn, and Zn remained high and above Swedish background values throughout the river and estuary.

### 3.3 Quantification of In‐Fauna

The sediment infauna collected at the estuary sites exhibited low abundance and diversity, with only two families of polychaetes (Maldanidae and Nereididae) and individuals from Echiuroidea and Sipuncula (Table S3). The two inner sites (E1 and E2) contained only individuals from the family Maldanidae, which were also found at E3 and E4, while Nereididae were present at E3–E5. Individuals of the family Echiuroidea were found at the more marine sites E4–E5, and the order Sipuncula was found only at E5.

### 3.4 Measurements of Estuarine Community Metabolism

**FIGURE 3:**
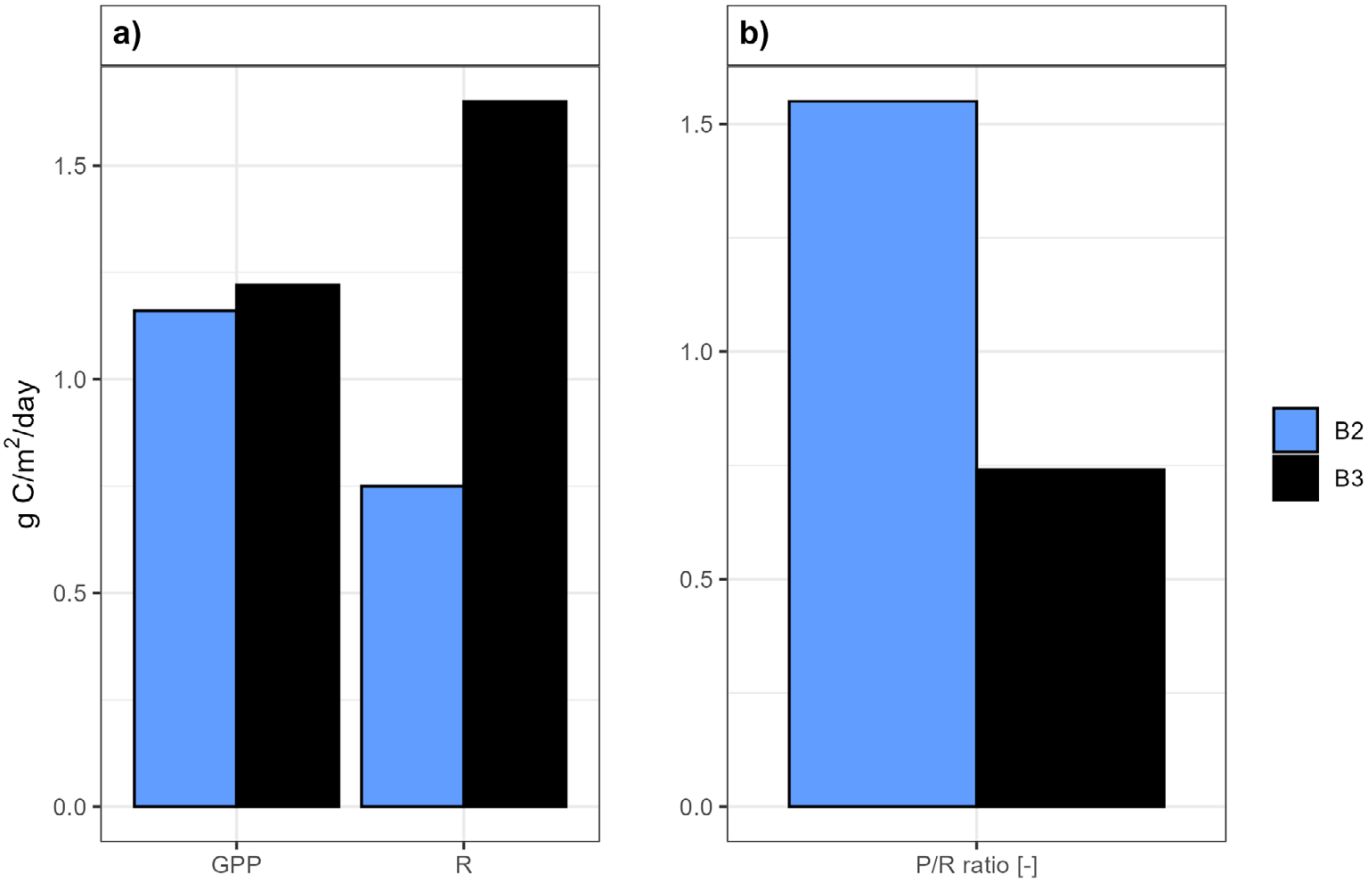
(a) Community metabolism at site B2 and B3, where C fluxes through respiration, R, and gross primary production, GPP, is presented per area per day. (b) The photosynthetic/respiratory (P/R) ratio indicates autotrophy at B2 and heterotrophy at B3.

There was a substantial difference in respiration between sites B2 and B3, with rates of 0.75 and 1.65 g C/m^2^ per day, respectively (Figure 3). Gross primary production was similar at the two sites, with 1.16 and 1.22 g C/m^2^ per day at B2 and B3, respectively (Figure 3). This relatively high primary production rate is likely due to the high abundance of cyanobacteria at these sites. Although site B3 exhibited slightly higher gross primary production, the resulting net production was negative due to high community respiration, indicating a greater abundance of heterotrophs and higher total bacterial biomass at this site. The daily P/R ratios were 1.55 and 0.74 for the two sites (Figure 3), indicating that on this date, B2 was dominated by autotrophic bacteria, while B3 was a heterotrophic community.

### 3.5 Bacterial Community Assessment Using Meta‐Barcoding

**FIGURE 4:**
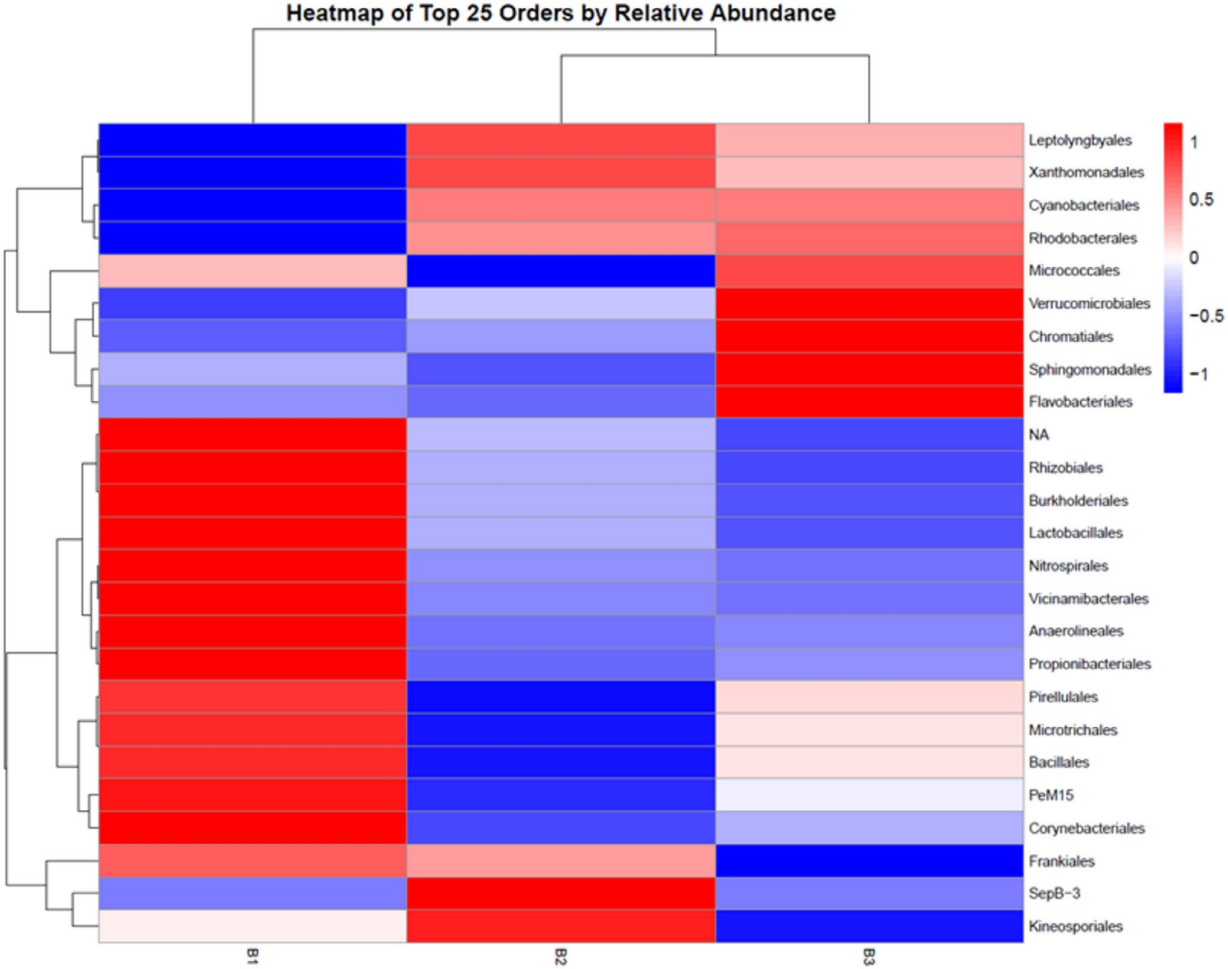
The heatmap illustrates the relative abundance of the top 25 microbial orders across three sampling sites (B1, B2, and B3). Relative abundance values are scaled by rows (orders), with red representing higher abundance, blue indicating lower abundance, and white showing intermediate abundance within each order. NA, sequence not found in the SILVA database.

Orders such as Leptolyngbyales, Xanthomonadales, and Cyanobacteriales showed consistently high relative abundance at sites B2 and B3, with Cyanobacteriales dominating (28% and 22% relative abundance, respectively, Figure 4). In contrast, Nitrospirales and Rhizobiales were more abundant at site B1. These differences suggest distinct microbial community structures among the sites.

Rhodobacterales and Micrococcales exhibited substantial variability in relative abundance across sites, with shifts of up to two orders of magnitude between sites B1, B2, and B3. Anaerolineales were notably more prominent at site B1, suggesting localized anoxic or hypoxic conditions favoring anaerobic bacteria. In contrast, anaerobic bacteria had very low relative abundances at sites B2 and B3 (Figure 4).

Clustering analysis of microbial composition revealed that sites B2 and B3 grouped closely, indicating similar environmental conditions. In contrast, site B1 formed a distinct cluster, reflecting differences in microbial order composition.

### 3.6 Toxicity Experiments on the Freshwater Amphipod *Hyalella azteca*

**FIGURE 5:**
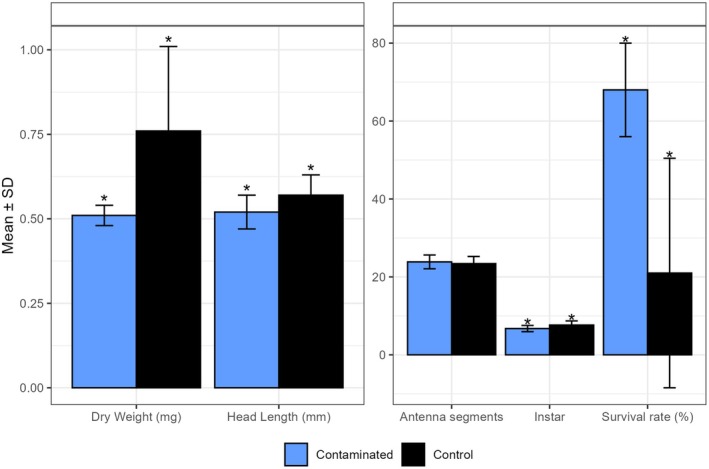
Mean values and *t*‐test analysis of morphological traits and survival of *Hyalella azteca* in contaminated and control conditions. Mean values are presented with standard deviation (SD). *Significant difference, significance level set to *p* < 0.05.

There were clear differences in the development of certain morphological traits of the *Hyalella azteca* individuals exposed to water and sediment from the ASS site compared to the control site. The head length was 0.52 mm in the contaminated water, compared to 0.57 mm in the control, indicating impaired development when exposed to ASS‐contaminated water (*p* < 0.001) (Figure 5, Table 2).

**TABLE 2:** Two‐Sample *t*‐tests Assuming Unequal Variances, (two‐tail). “*t* crit” = *t* critical value, “df” = degree of freedom.

Kolumn1	*t* ^a^	*t* crit	df	*p*
Head length (mm)	4.07	2.01	54	**< 0.001**
Dry weight (mg)	6.28	2.04	31	**< 0.001**
Antenna segments	0.31	2	63	0.3
Instar	4.07	2	54	**< 0.001**
Survival rate (%)	2.53	2.45	6	**0.045**

*Note:* Significant differences are indicated with bold *p*‐values.

^a^ Two‐Sample Assuming Unequal Variances.

Additionally, the dry weight was much lower in individuals exposed to contaminated water compared to the control (0.51 mg and 0.76 mg, respectively, *p* < 0.001) (Figure 5, Table 2). The instar, a measure of developmental stage in the life cycle, was more advanced in the control group (7.65) compared to contaminated conditions (6.75, *p* < 0.001) (Figure 5, Table 2).

The survival rate was higher under contaminated conditions, with 68% of individuals surviving through the experimental period, compared to 21% for the control site (*p* < 0.045) (Figure 5, Table 2). The number of antenna segments did not differ between treatments, with 23.4 in the control and 23.9 in the contaminated conditions (Figure 5, Table 2). The sex ratio was consistent across treatments, with 69% females and 31% males in both conditions.

The concentrations of metals in the sediment and water from the two sites are presented in Table S4. Almost all elements related to ASS were higher in the contaminated water and sediment compared to the control, except for Zn (in both water and sediment), Cu, and Cd (in sediment), which were higher at the control site. As and Pb were substantially higher at the control site, although these are elements not related to ASS.

## 4 Discussion

### 4.1 Pollution Source and Dispersion of Metals

Elevated concentrations of potentially harmful metals alongside reduced pH were observed in the agricultural ditch, riverine system, and estuary downstream of the study area, supporting the hypothesis that runoff from the agricultural ASS contributes metals to water, biota, and sediments in adjacent estuarine and marine environments.

The pH of the river outflow water from Ramsjö Canal was measured to be lower than that of eight other rivers in Halland County during March and October. However, in May, the Ramsjö site had a similar or even higher pH than that of the other rivers. These fluctuations might be explained by weather conditions—both winter 2020/21 and summer 2021 were wet but warm—and at the same time highlight the necessity of permanent but also event‐based sampling of ASS‐affected river systems. Although the pH was substantially lower in the agricultural ditch compared to the river mouth and estuary, the acidic effluents caused low pH levels at several river and estuary sites. At site E5, the pH was 8.1, considered normal for oceanic waters in non‐acidified conditions; hence, the acidic effect from ASS runoff did not seem to extend all the way out to the sea.

Several ASS‐related metals exhibited higher concentrations in the water of the agricultural ditch and, in most cases, throughout the estuary, compared to reference levels for rivers in the area (Herbert et al. 2009). Nyman et al. (2024) found that even small drops in groundwater levels could lead to significant mobilization of metals such as Al, Cd, Co, Mn, Ni, S, and Zn across Swedish coastal plains. Particularly concerning were the exceptionally high water‐soluble concentrations of Co, Mn, and Ni released from oxidized and incubated acid sulfate soil profiles, highlighting the threat posed by climate‐induced or anthropogenic drainage. Complementing these results, Hulisz et al. (2022) demonstrated that acid sulfate soils in the southern Baltic zone, even when relatively low in total metal content, released high proportions (up to 80%) of Cr, Cu, Ni, Pb, and Zn in mobile forms under both natural and artificial drainage conditions, emphasizing the leaching risk even in seemingly moderate ASS settings.

In addition to water, sediment analysis revealed significant enrichment of ASS‐derived metals, such as Al, Zn, and Co, in both the river and the estuary. Shahabi‐Ghahfarokhi et al. (2022) reported the formation of Fe‐rich precipitates in a channel collecting farm drains from ASS sites in southern Sweden, but many metals (including Mn, Zn, Ni Co, and Cd) appeared to bypass this retention and were inferred to disperse further downstream. Other studies have shown that ASS‐related metals typically have high concentrations in the inner parts of estuaries, especially near the river mouth, with concentrations rapidly decreasing with distance (Nordmyr et al. 2008; Nystrand et al. 2016). This trend was observed in the current study, where the highest concentrations of most ASS‐related metals were found near the river mouth in both water and sediment. This is likely due to the affinity of metals to bind with sediment particles or organic matter (Nordmyr et al. 2008), or the precipitation of metals when the low pH riverine water blends with more alkaline water in the estuary (Virtasalo et al. 2023) preventing them from remaining in the liquid phase and resulting in early capture in the sediment during discharge from the river. However, studies on the long‐term effects of ASS leachate report metal enhancement further out in the estuary and toward the Baltic Sea (Virtasalo et al. 2020; Dalhem et al. 2025).

Furthermore, at site E5, considered an oceanic location, the concentrations of certain ASS‐associated metals were considerably elevated relative to both historical Kattegat Sea values (Magnusson and Westerlund 1983) and environmental thresholds set by the Environmental Quality Standards Directive (EQSD 2013/39/EU). For example, Cu was measured at 28.8 μg/L, vastly exceeding the EQS for marine waters (1.3 μg/L). Similarly, Zn reached 7.6 μg/L, approaching the freshwater EQS threshold of 7.6 μg/L, while Ni at 4.0 μg/L remained below the marine EQS (8.6 μg/L) but may still pose a risk depending on its bioavailable fraction. Fe, though not regulated under EQS, was also markedly elevated (29.5 μg/L). In contrast, the concentrations in the Kattegat Sea upper water column were much lower: approximately 0.5 μg/L for Cu, 0.6 μg/L for Fe, and 0.7 μg/L for Zn (Herbert et al. 2009; Magnusson and Westerlund 1983). This indicates that metals from the ASS‐impacted areas are dispersing further into the system and may, therefore, cause detrimental effects not only on estuarine but also on marine life in the area. ASS leakage must therefore be considered more than a localized problem which requires both improved monitoring and integrated management approaches across land–water interfaces (Toivonen and Boman 2024).

In summary, the study clearly shows that the drainage from the agricultural ASS site increases acidity and metal concentrations in the riverine water of the area. This pollution is translocating from the source downstream to the estuary, acting as a sink of the riverine system. However, the results also show that metal dispersal is not confined to the river mouth and may extend further into the ocean.

### 4.2 Synergistic Effects of pH and Metal Toxicity

As hypothesized, the ASS‐related runoff adversely affected aquatic biota in the estuary, challenging efforts to maintain biodiversity and ecosystem health.

Exposed to water and sediment from the ASS area, the developmental stages of the freshwater amphipod *Hyalella azteca* were substantially impaired compared to control conditions. In the experimental conditions, most metals had higher concentrations in the contaminated water and sediment compared to the control, except for As, Fe, Pb (not related to ASS), and Zn (an ASS metal). Additionally, Cu concentrations were higher in the control sediment. These results indicate that the medium collected from the site not affected by ASS leakage still possessed potential toxic properties, which may affect the health of the test amphipods. Not only metals but more prominently, low pH have negative effects on *H. azteca* (Chapman et al. 2012). Additionally, low pH may transform metals into more toxic forms for invertebrates (Fornaroli et al. 2018; Jeong et al. 2023), and high heavy metal concentrations have less adverse effects on, for example, invertebrates (Jeong et al. 2023), diatoms, fish (Vehanen et al. 2022), and aquatic macrophytes (Öhlander et al. 2014) when pH is higher. Hence, the low pH from the ASS‐affected water may be the key factor driving the lowered vitality as it does have a direct negative effect on the test organisms but also enhance metal toxicity. Moreover, a low pH enhances Al hydroxide the dissolution and Al silicate weathering, which in turn promotes Al solubilization and transport (Fältmarsch et al. 2008). This combination has direct toxic properties, negatively affecting macrobenthos abundance (Corfield 2000; Fornaroli et al. 2018) and fish vitality (Authman et al. 2015; Sutela and Vehanen 2017). Notably, an Al concentration of 0.5 mg/L, a concentration frequently exceeded at the ditch and river sites, is considered lethal for several fish species and macroinvertebrates (Vuorinen et al. 1993; Corfield 2000). Additionally, avian and aquatic top predators from ASS‐affected areas have higher metal concentrations in their blood compared to animals from non‐ASS regions (Alhonen et al. 1997; Vainio et al. 2020). This suggests that bioavailability and biomagnification of metals may occur across multiple trophic levels, which could pose a potential risk to human health (Fältmarsch et al. 2008; Åström and Roos 2022). Hence, this highlights the problematic double‐edged threat to aquatic biota that ASS leakage imposes.

In control conditions, *Hyalella azteca* exhibited significantly longer head lengths, higher dry weights, and more pronounced instar stages, indicating more developed life stages. There were also more antenna segments in the control specimens. However, this anatomical feature is sensitive, and segments may have fallen off during the experimental and handling procedures, so results should be interpreted cautiously. Interestingly, the survival rate was higher in the contaminated water, suggesting that more resources may be allocated toward survival than growth and development. Upregulation of genes related to vital metabolic functions and stress responses when exposed to heavy metals (Poynton et al. 2018) may indicate this allocation. Several elements targeted in our study, including Zn, Ni, Pb, and Cd, were found to be far below the LC50 concentrations of these metals (Schubauer‐Berigan et al. 1993). Hence, the negative physiological responses could be attributed to the metal toxicity and the acidity of the contaminated water, although the concentrations were not high enough to promote a lethal effect. The results might thus indicate that, under normal development, mortality rates are relatively high for this species, and that impaired development under “non‐lethal exposure scenarios” reduces mortalities in the exposed population, something that might look like a paradox.

During the field visits, a low abundance of aquatic life was observed in the estuary when screened with an aquascope. In addition to the lack of fish in the river and estuary, which might be explained by low pH affecting fish mortality and reproductive success (Pinheiro et al. 2019; Toivonen et al. 2020), no bivalves were encountered, but numerous empty small blue mussel shells ( *Mytilus edulis* ) were found. Low pH can contribute to coastal acidification, with similar adverse effects on calcareous organisms as those observed in ocean acidification. These effects include reduced biomineralization, calcification, and survival rates in species such as oysters and mussels (Barton et al. 2015; Fitzer et al. 2018). Also, heavy metal exposure may impair bivalve vitality (e.g., Jeong et al. 2023). Whether this absence is directly related to ASS effluents has not been conclusively studied in the present survey. The same applies to the seemingly absent fish, where trial fishing and egg/larval recruitment tests would be required to confirm this hypothesis.

Further, the estuary showed limited submerged vegetation, with algal tissue samples for metal concentration analysis only present at sites E1–E3. At these sites, the amounts of ASS‐associated metals (Al Co, Mn, Ni, and Zn) were several times higher than those measured in green filamentous algae (*Cladophora* sp.) from a non‐ASS area near the Tjärnö Marine Laboratory on the Swedish west coast. For example, at site E1, Al concentrations reached 7.69 g/kg dry weight (DW), and at site E2, Zn concentrations were 130 mg/kg DW. In comparison, the Tjärnö specimens measured 0.369 g Al/kg DW and 22 mg Zn/kg DW. The only ASS‐related metal with higher content at Tjärnö was Cu (Olsson et al. 2020). Despite this, the Tjärnö samples exhibited similar or even higher amounts of other metals, including As, Cr, and Pb. Additionally, much lower concentrations of Cu, Mn, and Zn were found in Norwegian samples (7.0, 56, and 13 mg/kg DW, respectively; Biancarosa et al. 2018) compared to the current study. The high metal content in low trophic organisms, such as green filamentous algae, highlights their bioavailability and potential for biomagnification.

In contrast to the lack of visible and infaunal organisms, high microbial activity was detected in the sediment, as demonstrated by community metabolism measurements. At site B2, the net primary production (0.41 g C/m^2^ per day) exceeded the daily respiration rate, indicating an autotrophic state. The high gross primary production at sites B2 and B3 is likely attributed to the high abundance of cyanobacteria, as identified through metabarcoding, where the orders Leptolyngbyales and Cyanobacterales were found to be dominant. However, at site B3, community metabolism measurements indicated an overall net heterotrophic environment, which is a common state for estuarine waters globally (e.g., Cai 2011). The distinct clustered microbial community composition at sites B2 and B3 compared to B1 suggests that the distribution in the estuary is patchy, as the sampling points are geographically close. However, this patchiness may be related to small‐scale differences in environmental conditions as B1 was situated in a quite quiescent zone. Several taxa present in the estuary with relatively high abundance are associated with acidic conditions, e.g., Xanthomonadales [Rhodobacter] (Green et al. 2012) and Bryobacteraceae [Vicinamibacterales] (Dedysh 2019), and sulfur cycling, e.g., Rhodobacterales (Anderson and Harvey 2022; Rajeev and Cho 2024). Various families previously identified in Swedish or Finnish ASS soils were present in the estuary such as the Mn‐oxidizing Flavobacterium [Flavobacteriales] (Yu et al. 2024), and the semi‐acidophilic Gallionellacea, an important mediator for sulfur and iron cycling (Högfors‐Rönnholm et al. 2022) and reported specifically in this geographical region of Sweden (Johnson et al. 2024). However, Ktedonobacteraceae, the most common family in the oxidized zone in northern boreal areas (Johnson et al. 2024), was absent in the estuary. It remains unclear whether this supports the observed regional differences between northern and southern Sweden (Johnson et al. 2024) or whether it is instead a result of submerged hypoxic conditions in the estuary.

The low abundance of infaunal organisms, along with the poor representation of phylogenetic groups, suggests that the estuary is subjected to environmental conditions that are unfavorable for invertebrates, at least periodically. The only infaunal groups found were two polychaete families (Neridae and Maldanidae), as well as individuals from the Echiuroidae and Sipunculoid families, all of which are known to tolerate low oxygen conditions (Diaz and Rosenberg 1995). This observation suggests that the system experiences hypoxic conditions periodically, as supported by the presence of the Anaerolineales, which are typical members of anaerobic microbial communities, indicating localized anoxic or hypoxic conditions. Hence, the estuarine biological community appears to be at risk, in addition to the low pH and high metal concentration, of hypoxic conditions, further complicating its survival.

While not definitive scientific evidence, a group of local ornithologists reported a significant decline in birdlife, particularly waders, in the estuary in recent years. These observations suggest that the estuary may be a less productive habitat for aquatic organisms. Increased impact from recently developed sulfuric soils, leading to reduced infaunal and macrophyte abundance, could be driving the birds to seek food in other estuarine systems along the Swedish west coast. As Ramsjö canal flows into the Nature 2000 SPA Birds directive area “Galtabäck‐Lynga coastal meadows” (Länsstyrelsen 2025), it may be of high concern for local authorities to address this issue with high priority. Considering the observed shifts in seasonal groundwater recharge patterns in the geographical region (Nygren et al. 2020), continued or intensified leakage from ASS may pose an emerging environmental threat, one that warrants closer attention and future mitigation efforts at both local and national levels.

## 5 Conclusions

This study presents an initial report on the situation in an estuary affected by acid sulfate soils (ASS) in an area of Sweden where this has not been investigated before. Several aspects need to be considered in more detail with additional research. However, the study clearly states that (i) metals and acidity from the agricultural ASS are transported via riverine water and accumulate in the sediment downstream and biota in the estuary, (ii) this has adverse effects on aquatic flora and fauna, and (iii) elevated metal concentrations in the sediment may serve as long‐term pollution sources for the aquatic system.

These findings underscore the need for targeted mitigation strategies to address ASS impacts, particularly in regions where ASS effluents have not been comprehensively managed. The results also raise concerns regarding compliance with the EU Water Framework Directive, as ASS effects pose a significant challenge to achieving ecological and chemical water quality objectives in affected areas. Further research is recommended to explore long‐term impacts on biodiversity, biogeochemical cycles, and potential remediation strategies.

## Author Contributions

**Lina M. Rasmusson:** conceptualization (equal), data curation (equal), formal analysis (equal), funding acquisition (equal), investigation (equal), methodology (equal), project administration (equal), resources (equal), supervision (supporting), visualization (supporting), writing – original draft (lead), writing – review and editing (lead). **Markus Giese:** conceptualization (equal), data curation (equal), formal analysis (equal), funding acquisition (lead), investigation (equal), methodology (equal), project administration (equal), resources (equal), visualization (supporting), writing – original draft (supporting), writing – review and editing (equal). **Michael Tedengren:** conceptualization (supporting), formal analysis (supporting), investigation (supporting), methodology (supporting), resources (equal), supervision (lead), writing – review and editing (equal). **Nathalie Strokirk:** data curation (equal), formal analysis (equal), investigation (lead), methodology (equal), visualization (equal), writing – original draft (supporting), writing – review and editing (equal).

## Conflicts of Interest

The authors declare no conflicts of interest.

## Supporting information


Data S1.

## Data Availability

All data are available in the manuscript or Supporting Information except raw data for laboratory experiments and metabarcoding that is available through FigShare at https://doi.org/10.6084/m9.figshare.28239704.
