# Supplementary material for: Acid Sulfate Soils and Their Pathways of Impact: A Swedish Case Study
Source: Ecol Evol. 2025 Jul 10;15(7):e71732. doi: 10.1002/ece3.71732 (PMC12243068; doi:10.1002/ece3.71732)
Supplement: Supplementary file 1 — Data S1. [file ECE3-15-e71732-s001.docx]

**SUPPLEMENTARY INFORMATION**

| **Sample point** | **Coordinates** | **Time** | **Point M** | **Water** | **Sediment** | **Tissue** | **IF** | **Bacteria** | **CM** |
| --- | --- | --- | --- | --- | --- | --- | --- | --- | --- |
| Ditch (D) | 56°56'44.4"N 12°25'30.7"E | March | X | X |  |  |  |  |  |
|  |  | May | X | X | X |  |  |  |  |
|  |  | October | X | X |  |  |  |  |  |
| River 1 (R1) | 56°59'02.3"N 12°21'30.0"E | March | X |  |  |  |  |  |  |
|  |  | May | X | X | X |  |  |  |  |
|  |  | October | X | X |  |  |  |  |  |
| River 2 (R2) | 56°59'06.0"N 12°21'24.0"E | March | X | X |  |  |  |  |  |
|  |  | May | X | X | X |  |  |  |  |
|  |  | October | X | X |  |  |  |  |  |
| Estuary 1 (E1) | 56°59'04.5"N 12°21'23.6"E | May | X | X | X | X | X |  |  |
| Estuary 2 (E2) | 56°59'05.5"N 12°21'21.4"E | May | X | X | X | X | X |  |  |
| Estuary 3 (E3) | 56°59'06.4"N 12°21'18.9"E | May | X | X | X | X | X |  |  |
| Estuary 4 (E4) | 56°59'07.4"N 12°21'16.6"E | May | X | X | X |  | X |  |  |
| Estuary 5 (E5) | 56°59'08.4"N 12°21'14.4"E | May |  | X | X |  | X |  |  |
| Bacteria 1 (B1) | 56°59'04.1"N 12°21'24.9"E | July |  |  |  |  |  | X |  |
| Bacteria 2 (B2) | 56°59'04.2"N 12°21'24.5"E | July |  |  |  |  |  | X | X |
| Bacteria 3 (B3) | 56°59'04.3"N 12°21'24.4"E | July |  |  |  |  |  | X | X |

**Table S1.** *Overview of when (month), where (sampling points presented in Figure 1) and what have been measured or collected during the field campaigns. Dates for field sampling March 2^nd^, May 17-20^th^, July 3^rd^ and October 11-14^th^ Point M=point measurements IF=Infauna CM=community metabolism*

**Table S2.** *Water parameters measured in-situ at the different sample points (see Figure 1 and Table S1 for positions)*

| **Sample point** | **Month** | **°C** | **pH** | **EC [µS/cm]** |
| --- | --- | --- | --- | --- |
| **D** | March | 3.6 | 5.22 | 531 |
|  | May | 14.7 | 4.96 | 553 |
|  | October | 10.9 | 4.8 | 557 |
| **R1** | March | 3.7 | 6.89 | 425 |
|  | May | 10.7 | 7.11 | 408 |
|  | October | 10.8 | 6.73 | 420 |
| **R2** | March | 3.6 | 6.75 | 424 |
|  | May | 10.8 | 7.04 | 408 |
|  | October | 10.8 | 6.72 | 420 |
| **E1** | May | 12.6 | 7.16 | 427 |
| **E2** | May | 16.2 | 7.52 | 438 |
| **E3** | May | 12.6 | 7.4 | 800 |
| **E4** | May | 15.7 | 7.85 | 15 000 |
| **E5** | May | 14.4 | 8.12 | 22 000 |

***Table S3.*** *The infauna, only represented by two families of Polychaeta (Maldanidae and Nereididae), and the families Echiuroidea and Sipuncula presented with number of individuals per family per site (E1-E5), and recalculation of number of individuals per m^2^ in brackets.*

| **Site** | **Nereididae** | **Maldanidae** | **Echiuroidae** | **Sipuncula** |
| --- | --- | --- | --- | --- |
| **E1** |  | 4 [249] |  |  |
| **E2** |  | 2 [124] |  |  |
| **E3** | 2 [124] | 1 [62] |  |  |
| **E4** | 1 [62] | 2 [124] | 1 [62] |  |
| **E5** | 2 [124] |  | 1 [62] | 1 [62] |

**Table S4.** *Element concentrations in sediment and water from the laboratory experiments for the contaminated and control site. Sediment concentration = mg/kg DW, water concentrations = µg/L (* = mg/L). Bold letters indicate elements related to acid sulfate soils. N/A = not applicable*

|  | **Sediment** | | **Water** | |
| --- | --- | --- | --- | --- |
|  | **Contam.** | **Control** | **Contam.** | **Control** |
| **Al** | 54 000 | 7120 | 21.7 | 3.1 |
| As | 5.7 | 4.1 | 0.47 | 0.54 |
| Ba | 57 | 63.1 | 32.1 | 20.5 |
| Ca | N/A | N/A | 38.6* | 36.2* |
| **Cd** | 0.56 | 0.59 | 0.0048 | <0.002 |
| **Co** | 11.5 | 4.6 | 0.084 | <0.005 |
| Cr | 28 | 21.2 | 0.16 | 0.028 |
| **Cu** | 42.9 | 128 | 91.5 | 46 |
| **Fe** | N/A | N/A | 0.013* | 0.014* |
| Hg | <0.2 | 0.34 | <0.002 | <0.002 |
| K | N/A | N/A | 6.5* | 3.5* |
| Mg | N/A | N/A | 10.3* | 8.7* |
| **Mn** | N/A | N/A | 0.65 | 0.2 |
| Mo | N/A | N/A | 1.15 | 0.27 |
| Na | N/A | N/A | 35.5* | 53.4* |
| **Ni** | 25 | 12.8 | 2.3 | 0.37 |
| P | N/A | N/A | 19.8 | 27.1 |
| Pb | 13.4 | 149 | <0.01 | 0.023 |
| Si | N/A | N/A | 6.03* | 0.25* |
| **Sr** | N/A | N/A | 176 | 140 |
| V | 48 | 27.3 | 0.48 | 0.073 |
| **Zn** | 91.2 | 167 | 0.44 | 2.6 |
